# Temporal recalibration of vision

**DOI:** 10.1098/rspb.2010.1396

**Published:** 2010-09-08

**Authors:** Derek H. Arnold, Kielan Yarrow

**Affiliations:** 1 School of Psychology, The University of Queensland, Brisbane, QLD 4055, Australia; 2 Department of Psychology, City University London, London, UK

**Keywords:** timing perception, perceptual binding, colour, motion

## Abstract

Our sense of relative timing is malleable. For instance, visual signals can be made to seem synchronous with *earlier* sounds following prolonged exposure to an environment wherein auditory signals *precede* visual ones. Similarly, actions can be made to seem to precede their own consequences if an artificial delay is imposed for a period, and then removed. Here, we show that our sense of relative timing for combinations of visual changes is similarly pliant. We find that direction reversals can be made to seem synchronous with unusually early colour changes after prolonged exposure to a stimulus wherein colour changes precede direction changes. The opposite effect is induced by prolonged exposure to colour changes that lag direction changes. Our data are consistent with the proposal that our sense of timing for changes encoded by distinct sensory mechanisms can adjust, at least to some degree, to the prevailing environment. Moreover, they reveal that visual analyses of colour and motion are sufficiently independent for this to occur.

## 1. Introduction

Cross-modal temporal recalibration refers to a shift in the point of subjective simultaneity (PSS) between two types of event following prolonged exposure to asynchronous inputs. For instance, repeated exposures (adaptation) to sights that *lead* corresponding sounds can shift the PSS for vision and audition towards earlier sights. Conversely, adaptation to sights that lag sounds can have the opposite effect [1–3]. These data seem to suggest that our sense of relative timing can adjust according to the prevailing environment. Thus if sounds typically lag sights in the environment, this relationship will be judged as typical, and therefore as synchronous. Moreover, if the environment changes, such that sounds are made to lead sights, this new relationship will be judged as typical and therefore as synchronous.

Cross-modal temporal recalibration has been demonstrated for all combinations of signals paired from vision, audition and touch [4,5]. However, it is unclear if temporal recalibration can be observed for events encoded within a single sensory modality.

Temporal recalibration necessitates that the timing of the affected events be encoded somewhat independently, thereby allowing for relative shifts. Combinations of events from separate sensory modalities might therefore be ideally suited. However, there is ample evidence for functional specialization *within* the human visual system. For instance, distinct cortical structures seem to be specialized for the analysis of different stimulus properties, such as colour and movement [6,7]. The fact that these functionally specialized areas are located in different regions of the brain is presumably a prerequisite for the selective visual deficits that can arise following focal brain damage. Such deficits have been documented for both colour [8] and motion perception [9]. However, while there is ample evidence for independent coding of colour and motion, it is also well established that at least some visual neurons are sensitive to *both* colour *and* direction of motion [10–12]. Thus, it is unclear whether analyses of colour and motion are sufficiently independent to allow for adaptation-induced shifts in relative timing.

While there is some debate concerning whether functional specialization within the human visual system [7] creates a need for integrative processes [11], several perceptual phenomena seem to confirm this necessity. For instance, when a stimulus contains multiple colours and directions of motion, perceptual pairings of colour and motion can be systematically and persistently erroneous [13]. This not only confirms the existence of an integrative process, by which colour and motion signals are combined to form a coherent whole, but establishes that this process is error prone. Our sense of relative timing for colour and direction changes is similarly susceptible to error, with colour changes often seeming to precede physically synchronous direction changes ([14–18]; but see [19]).

The independence of colour and motion processing suggested by previous studies [8,9,13,17,18] encouraged us to examine the possibility that our sense of timing for these attributes might, at least to some extent, adjust according to the dynamics of the contemporary environment. The results of the following experiment answer this question in the affirmative.

## 2. Methods

Stimuli were generated using Matlab software to drive a VSG 2/3F stimulus generator (Cambridge Research Systems) and were displayed on a gamma-corrected Sony Trinitron G420 monitor at a resolution of 1024 × 768 pixels and a refresh rate of 120 Hz. All stimuli were viewed from 57 cm, with the observer's head restrained by a chinrest.

**Figure 1.**
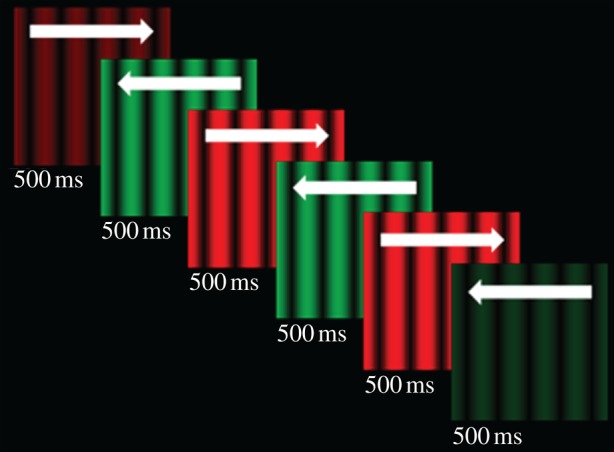
Depiction of a test stimulus presentation. The stimulus oscillated in colour (green/red) and direction of motion (left/right). The peak luminance of the stimulus was linearly ramped on and off at the beginning and the end of each test presentation (for 500 ms). Here, we have depicted a situation wherein changes in direction and colour were physically synchronous, but in most trials colour changes were offset relative to direction changes. We have also depicted a situation wherein the start of the animation cycle is synched with the start of the test presentation. However, the starting point, in terms of the animation cycle, was determined at random on a trial-by-trial basis.

The stimulus, depicted in figure 1*a*, consisted of a sinusoidal luminance-modulated grating with a Michelson contrast of 100 per cent and a spatial frequency of one cycle per degree of visual angle presented against a black background. The grating waveform drifted at a rate of 10 Hz, and reversed direction (left/right) at a periodicity of 1 Hz. The grating also alternated in colour at a rate of 1 Hz, between a peak red (CIE *x* = 0.614, *y* = 0.342, *Y* = 17) and peak green (CIE *x* = 0.276, *y* = 0.607, *Y* = 17). This basic stimulus was used as both an adapting and test stimulus.

There were three types of runs of trials. In baseline runs of trials, participants simply viewed and made judgments of test stimuli. During adaptation runs of trials, participants passively viewed adapting stimuli prior to each test stimulus presentation. On the first trial, the adapting stimulus presentation persisted for 45 s, whereas on most subsequent trials the adapting stimulus presentation persisted for 6 s. There was, however, another 45 s presentation of the adapting stimulus on the 33rd trial during each adaptation run of trials. There was a one-second blank ISI between each adaptation and test stimulus presentation. During separate runs of trials, colour changes in the adapting stimulus *either* preceded (colour lead) or succeeded (colour lag) direction changes by 200 ms. All other features of the adapting stimulus animation were as for the test stimulus.

Each presentation of the test stimulus persisted for 3 s. The luminance of the test stimulus was linearly ramped on and off, from black, for 500 ms at the start and the end of each test presentation—so the test stimulus seemed to fade in and out. Participants saw two cycles of the test stimulus animation at full luminance on each trial. The starting point, in terms of the test stimulus animation cycle, was randomized on a trial-by-trial basis. After viewing each test stimulus participants were required to indicate if the colour and direction changes had been synchronous or asynchronous.

During each run of trials, the physical timing of test colour changes, relative to direction changes, was manipulated (±250 ms) according to the method of constant stimuli. A complete run of trials involved six presentations of 11 colour/direction change timing relationships, 66 trials in total.

There were five observers, all naive as to the purpose of the study. On separate days, each observer completed two baseline runs of trials and then two adaptation runs of trials, with colour changes that either preceded or lagged direction changes. The order in which the adaptation conditions were completed was determined in a pseudo-random fashion, with two observers completing the colour leading condition and then the colour lagging. Other observers completed the conditions in the reverse order.

**Figure 2.**
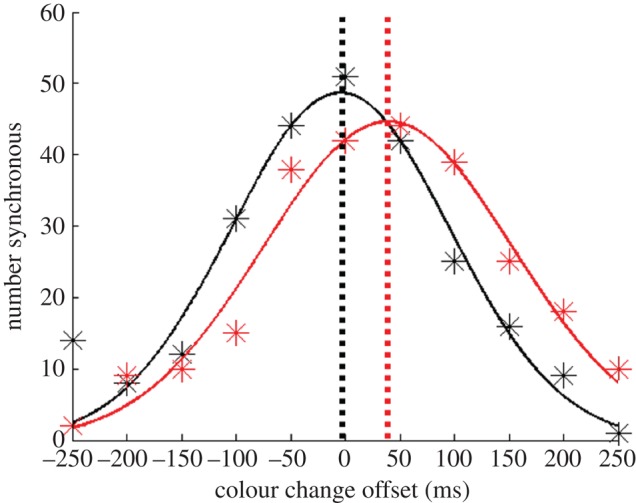
Gaussian functions fitted to distributions of reported colour/motion synchrony, summed across all participants, as a function of the physical timing of colour changes relative to direction changes. Direction changes occurred at 0 ms. Data are derived from trial runs involving adaptation to colour changes that *preceded* (black) and lagged (red) direction changes by 200 ms. These data depictions are provided for illustrative purposes only. Statistical analyses were based on individual data fits, with differences taken from PSS estimates from runs of trials with and without adaptation.

Data from each run of trials provided a distribution of perceived colour/direction change synchrony as a function of physical relative timing. Data from the two baseline runs of trials were collated and compared with data from the subsequent two adaptation runs of trials completed by the same observer. Gaussian functions were fitted to these data, and the peaks of the fitted functions taken as individual estimates of the PSS for colour and direction changes during the relevant runs of trials (figure 2).

## 3. Results

**Figure 3.**
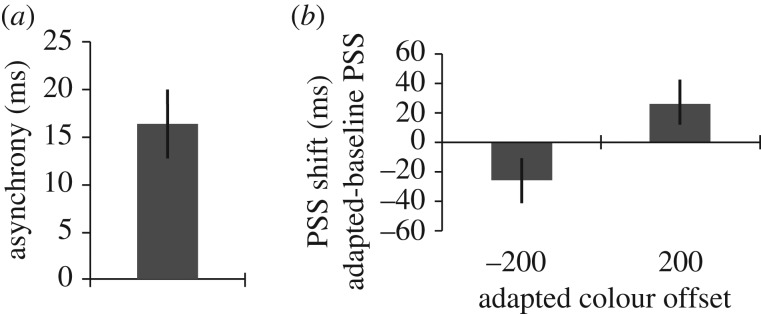
Bar graphs depicting the results of this study. Error bars denote standard error. (*a*) Colour/motion perceptual asynchrony in baseline runs of trials. (*b*) Shift in the PSS for colour and direction of motion changes, from baseline, after adaptation to colour changes that led (−200 ms) and lagged (+200 ms) colour changes.

In figure 3*a*, we have depicted the average PSS for colour and direction changes during baseline runs of trials. These data show that, in the absence of adaptation, colour changes seemed to be coincident with direction changes when they *lagged* the direction changes by approximately 19 ms (±5, *t*_4_ = 4.54, *p* = 0.01).

In figure 3*b*, we have depicted the magnitudes of PSS shifts following adaptation to colour changes that preceded and lagged direction changes by 200 ms. Shifts were calculated relative to the immediately preceding baseline sessions. In both cases adaptation shifted the PSS *towards* the adapted relationship resulting in substantial differences between the PSS shifts following adaptation to leading (−26 ms ± 13) and lagging (26 ms ± 13) colour changes (paired *t*_4_ = 3.49, *p* = 0.025).

## 4. Discussion

Our data show that the apparent relative timing of colour and direction changes is malleable. Prolonged exposure, i.e. adaptation, to temporally offset colour and direction changes shifted the PSS for these changes towards the adapted relationship. This suggests that our sense of timing for different visual events is shaped by the dynamics of the prevailing environment. If colour changes are made to lag direction changes, this relationship will begin to seem normal and therefore synchronous. The reverse situation can be induced by making colour changes lead direction changes.

Our data are consistent with recent studies concerning the apparent timing of changes encoded in different sensory modalities [1–5]. Here too the apparent timing of different stimulus changes can be drawn towards an adapted relationship. Presumably this type of relative shift is only possible because the apparent timing of the affected changes is encoded somewhat independently. Our data, therefore, demonstrate that timing codes for visual colour and direction changes are sufficiently independent to allow for relative shifts of perceived timing.

Our baseline trials reflect the apparent timing of colour and direction changes without adaptation, and are consistent with colour changes seeming to coincide with physically delayed (approx. 19 ms) direction changes. Larger colour advantages (approx. 100 ms) have been suggested by studies asking people to judge if a specific colour is predominantly paired with one direction of motion or another [17,18]. However, the magnitude of this effect is variable [15] and task dependent. For instance, one study asked people to judge the order in which colour and direction changes occurred, and found no evidence of an advantage for colour [16]. Other studies have used tasks like ours (asking people if colour and direction changes were synchronous) and have found robust advantages for colour [14,19]. Reaction times to changes in colour and direction also reflect a small advantage for colour [20]. Our baseline data are thus broadly consistent with the literature on perceived timing for colour and direction changes, which reflects a high degree of independence, with physically asynchronous inputs often judged as synchronous.

While our data are consistent with the impact of adapting to combinations of temporally offset cross-modal events, with adapted offsets beginning to look more synchronous than they did previously [1–3], there is an apparently opposite effect. When examining the timing of tactile events, participants seem to incorporate the statistics of prior exposures, which is referred to as a process of ‘Bayesian calibration’ [21]. In this case it would appear that experience of the offset between the adapted pair of tactile events is unchanged. Instead, other timing relationships, that had seemed synchronous in the absence of adaptation, are interpreted as being *like* the adapted pair—asynchronous [21]. We are not sure why exposure to offset tactile events results in this reversed temporal distortion. One possibility is that a Bayesian calibration will take place when making intra-modal judgments about identical features (e.g. changes in intensity at different spatial locations). However, our data concerning adaptation to an asynchrony signalled via distinct visual changes dictates that the discrepancy cannot be a necessary consequence of adapting and testing within a single sensory modality.

Given the functional architecture of sensory processing, determining whether perceptual events are synchronous or asynchronous is a non-trivial problem. A single physical event can be encoded within initially independent sensory systems, each marked by discrepant and variable processing speeds [22,23]. Taking vision as an example, it is apparent that this type of dilemma is not the special preserve of cross-modal perception, as different features of a single visual object can be encoded independently, and at different rates, by distinct visual mechanisms [24,25]. How then do we determine if different sensory events have occurred synchronously or asynchronously?

The existence of temporal recalibration suggests that one strategy, used to cope with the uncertainty caused by the variable time courses of processing in independent sensory systems, is to treat temporally proximate events that have *reliably* been presented at a specific timing relationship as being synchronous. Thus, perceived timing would not simply result from the physical timing of events, or from the time course of corresponding neural activity (although this factor is clearly important, e.g. [15,26,27]), but also from an experienced-based appraisal of the timing relationship between presented events. Our data reveal that this experienced-based influence on timing perception is not restricted to cross-modal perception. Rather, we have established the existence of an experience-based analysis that shapes our sense of timing for events encoded in a single sensory modality.
